# Evaluating the dissemination and scale-up of two evidence-based parenting interventions to reduce violence against children: study protocol

**DOI:** 10.1186/s43058-020-00086-6

**Published:** 2020-12-07

**Authors:** Yulia Shenderovich, Catherine L. Ward, Jamie M. Lachman, Inge Wessels, Hlengiwe Sacolo-Gwebu, Kufre Okop, Daniel Oliver, Lindokuhle L. Ngcobo, Mark Tomlinson, Zuyi Fang, Roselinde Janowski, Judy Hutchings, Frances Gardner, Lucie Cluver

**Affiliations:** 1 Department of Social Policy and Intervention, University of Oxford, Oxford, UK; 2 Department of Psychology, University of Cape Town, Cape Town, South Africa; 3 MRC/CSO Social and Public Health Sciences Unit, University of Glasgow, Glasgow, Scotland; 4 Catholic Relief Services, Baltimore, USA; 5 Clowns Without Borders South Africa, Cape Town, South Africa; 6 Institute for Life Course Health Research, Department of Global Health, Stellenbosch University, Stellenbosch, South Africa; 7 School of Nursing and Midwifery, Queens University, Belfast, UK; 8 School of Psychology, Bangor University, Bangor, Wales; 9 Department of Psychiatry and Mental Health, University of Cape Town, Cape Town, South Africa

**Keywords:** Evidence-based practice implementation, Parenting, Violence against children, Scale-up, Dissemination

## Abstract

**Background:**

Eliminating violence against children is a prominent policy goal, codified in the Sustainable Development Goals, and parenting programs are one approach to preventing and reducing violence. However, we know relatively little about dissemination and scale-up of parenting programs, particularly in low- and middle-income countries (LMICs). The scale-up of two parenting programs, Parenting for Lifelong Health (PLH) for Young Children and PLH for Parents and Teens, developed under Creative Commons licensing and tested in randomized trials, provides a unique opportunity to study their dissemination in 25 LMICs.

**Methods:**

The Scale-Up of Parenting Evaluation Research (SUPER) study uses a range of methods to study the dissemination of these two programs. The study will examine (1) process and extent of dissemination and scale-up, (2) how the programs are implemented and factors associated with variation in implementation, (3) violence against children and family outcomes before and after program implementation, (4) barriers and facilitators to sustained program delivery, and (5) costs and resources needed for implementation.

Primary data collection, focused on three case study projects, will include interviews and focus groups with program facilitators, coordinators, funders, and other stakeholders, and a summary of key organizational characteristics. Program reports and budgets will be reviewed as part of relevant contextual information. Secondary data analysis of routine data collected within ongoing implementation and existing research studies will explore family enrolment and attendance, as well as family reports of parenting practices, violence against children, child behavior, and child and caregiver wellbeing before and after program participation. We will also examine data on staff sociodemographic and professional background, and their competent adherence to the program, collected as part of staff training and certification.

**Discussion:**

This project will be the first study of its kind to draw on multiple data sources and methods to examine the dissemination and scale-up of a parenting program across multiple LMIC contexts. While this study reports on the implementation of two specific parenting programs, we anticipate that our findings will be of relevance across the field of parenting, as well as other violence prevention and social programs.

Contributions to the literature

- We describe a plan for learning from the widespread dissemination and scale-up of two parenting programs in a range of low- and middle-income country settings. Few previous studies have explored this scale of dissemination of a social program.
- The study will combine primary qualitative and organizational data, as well as secondary quantitative data collected by implementing agencies and other research teams.
- The results will provide insights into dissemination and implementation across the implementation stages identified in the Exploration, Preparation, Implementation and Sustainment framework.

## Background

The United Nations Sustainable Development Goal 16 has set the aspiration to “end abuse, exploitation, trafficking and all forms of violence against and torture of children” (target 16.2). This is no small challenge: over a billion children each year experience some form of violence, with most of the burden in low- and middle-income countries (LMICs) [1, 2]. Parenting programs are one of the seven strategies to prevent and reduce violence against children identified in the INSPIRE guidelines led by the World Health Organization (WHO) [3–5]. Parenting programs involve group-based or individual family meetings that aim to build effective parenting practices, strengthen positive parenting, reduce harsh or violent parenting, and improve child outcomes. Delivery of parenting programs may also help address multiple global goals such as reducing preventable deaths of infants and children under age five (target 3.2), reducing substance use (target 3.5), and violence (target 16.1) among children and young people, improving child’s and caregiver’s mental health (target 3.4), and promoting education (targets 4.1) [6–9].

There is growing interest in the scale-up of parenting programs [10–12]. Scale-up can be conceptualized as reaching wider geographical areas and more people, as well as embedding delivery into lasting systems, for instance, by integrating new programs into existing service systems [13–17]. In the field of parenting programs, as in other areas, many research-informed initiatives are not taken up widely [18, 19]. When programs are indeed taken up, several questions arise. One frequent concern is whether transporting a program from one cultural context to another may reduce family engagement and program effectiveness [20]. A recent systematic review found that parenting programs may have comparable or even greater effectiveness when implemented in regions and with populations different from where they were developed, with relatively minimal content adaptation [21, 22]. Yet not all transported family programs show effects [23, 24]. Another key question is whether program effects achieved in research trials can be replicated in routine services [25, 26]. Studies of parenting programs implemented across entire areas have shown that establishing and maintaining quality delivery is a challenge if programs are delivered by overburdened volunteers or staff [27–30].

Parenting for Lifelong Health (PLH) is an initiative led by individuals from the WHO, UNICEF, and the Universities of Bangor, Cape Town, Oxford, and Stellenbosch. It aims to develop, test, and scale-up parenting programs to reduce violence against children and improve child wellbeing in LMICs. Since starting work in 2012, PLH supported a suite of low-cost, Creative Commons-licensed parenting programs, including developing PLH for Young Children (2 to 9 years), and PLH for Parents and Teens (10 to 17 years). Both programs are group-based and can be supplemented with home visits. Group sessions normally take place in community venues, such as village halls. Initially tested in randomized controlled trials in South Africa with promising results [31–33], the programs have subsequently been tested by the developers and other researchers in multiple countries (e.g., the Philippines and Thailand; [34–36]). Program manuals are freely available online [37].

These two programs have experienced rapid dissemination in over 25 LMICs in sub-Saharan Africa, Eastern Europe, Southeast Asia, and the Caribbean (Czech Republic, Democratic Republic of the Congo, Federal Democratic Republic of Ethiopia, Kingdom of Eswatini, Kingdom of Lesotho, Kingdom of Thailand, Malaysia, Montenegro, Republic of Botswana, Republic of Cameroon, Republic of Côte d’Ivoire, Republic of Haiti, Republic of India, Republic of Kenya, Republic of Malawi, Republic of Moldova, Republic of North Macedonia, Republic of South Africa, Republic of South Sudan, Republic of the Philippines, Republic of Uganda, Republic of Zambia, Republic of Zimbabwe, Romania, and United Republic of Tanzania).

Delivery has been led by non-governmental organizations working with varying numbers of families. For example, in South Sudan, delivery by Catholic Relief Services reached several hundred families in 2017–2018 [38], while Pact Tanzania worked with 16,000 families in 11 districts in 2018–2019 and plans to work with approximately 47,800 families in 2020–2021 in 8 districts of Tanzania. In several countries, including Montenegro, South Africa, the Philippines, and Thailand, governments have led delivery. In many cases, PLH programs have been integrated into packages of services, such as the existing government conditional cash transfer system in the Philippines [34]. Adaptations of the programs have been tailored for a variety of populations, such as families reunited after children exit residential care in Kenya, and families with orphans and vulnerable children as well as families of adolescent girls in the context of HIV prevention in Tanzania.

In 2020, as part of the COVID-19 response, core program components were converted into tip sheets for families with an interagency collaboration including the WHO, UNICEF, UNODC, USAID, the Centers for Disease Control and Prevention, and the Global Partnership to End Violence Against Children, as well as NGOs [39]. Focusing on parenting during lockdown and school closures, the tips have been disseminated to an estimated over 72 million families in 178 countries.

Two non-profit organizations, Clowns Without Borders South Africa (CWBSA), and the Children’s Early Intervention Trust (CEIT) in Wales provide the main support in Africa and Europe, respectively. PLH uses a cascading dissemination model in which CWBSA and CEIT (i.e., the program purveyors [40]) transfer technical capacity to implementing agencies. Agencies interested in implementing PLH typically request technical support, which includes the following:

1. Adapting the programs to fit the local context and culture;
2. Conducting an implementation readiness assessment;
3. Providing materials and tools for implementation, monitoring, and evaluation;
4. Training frontline service providers including program facilitators, coaches, trainers, and coordinators;
5. Assessing and certifying personnel.

PLH facilitators range from community volunteers to professional psychologists depending on the context. Facilitators deliver the program to families—caregivers only for PLH for Young Children, and caregivers and their teens for PLH for Parents and Teens—and receive ongoing supportive supervision during delivery. To ensure high-quality program delivery, facilitator certification is done via structured live or video observations and is a requirement to be eligible to be trained as a PLH coach; coaches (usually professionals) provide ongoing support to facilitators. Likewise, PLH coaches are assessed for certification, which is a requirement before being trained as a PLH trainer who is licensed to train facilitators and coaches.

The global uptake of the PLH programs provides an unprecedented opportunity to explore questions of scale-up across multiple LMICs. As a blueprint for the Scale-Up of Parenting Evaluation Research (SUPER) study, this paper will (1) describe research questions and their rationale, (2) outline study structure and methods, and (3) discuss challenges and opportunities.

## Research questions

The study will use the Exploration, Preparation, Implementation and Sustainment (EPIS) framework [41, 42]. EPIS is a comprehensive model of implementation stages and their determinants. It organizes the study of implementation into the following stages: (1) exploration—the process of intervention selection; (2) preparation—setting up for implementation; (3) implementation—delivery of evidence-based practices and ongoing monitoring of the implementation process; and (4) sustainment—the process of program embedding in ongoing services. We use these stages to inform data collection, and analysis—for instance, to structure our key informant interview and focus group questions.

The SUPER study focuses on the following research questions.

### *(1) What is the process and extent of dissemination and scale-up of PLH programs?*

Dissemination of evidence-informed practices is at the heart of implementation research. It is important to understand how and why PLH has been taken up by funders and implementers since this will inform dissemination of similar programs in future and of the same programs in new settings [18]. The dissemination approach may also influence the quality and sustainment of the program. We will map the types of organizations, contexts, populations, and service combinations where PLH has been adopted, as well as identifying several cases where it was considered but not taken up.

#### (2) How are the PLH programs implemented in various contexts and what are the factors associated with variation in implementation? 

Program fidelity and adaptation exist in constant tension [43]. While strict adherence to protocols may ensure fidelity to the program as it was originally tested, adaptation is often considered necessary to fit new settings and populations [44]. In addition to formal systematic adaptation, adaptation often occurs during delivery in a more ad hoc, informal manner. Facilitators may change session content to address the concerns of the families with whom they work. Due to external factors, such as funding cycles or agricultural seasons, implementation agencies may adjust intervention length or facilitator recruitment processes. This cannot only lead to intervention drift [45] but also to innovations and maturation that could strengthen program outcomes [46]. During the COVID-19 pandemic, PLH adaptations have included remote training of implementation staff and remote provision of family support instead of in-person. To understand how PLH is implemented at scale, we will examine data on program delivery, adaptation, and participant engagement, and factors associated with these implementation outcomes.

#### (3) Are there changes in violence against children and other targeted family outcomes, following program delivery? 

Previous studies of parenting programs within routine delivery have examined family outcomes using various data sources. These include examining administrative data from social services in the USA [28], data collected by teachers in Scotland [29], families completing questionnaires in England [47], and data collected by research assistants in Kenya from several hundred families [48]. These studies have found mixed results in terms of changes in family outcomes. To the best of our knowledge, no large-scale data analysis of family outcomes has been reported in LMICs during ongoing parenting program delivery. We will conduct a secondary analysis of family-level pre-post outcome data collected by implementing partners in an estimated 5–8 countries through existing monitoring systems.

#### (4) What are barriers and facilitators to sustainment of PLH programs? 

Since the two PLH programs have been implemented for only a few years, it is important to examine the prospects and challenges for sustainment, such as via integration into existing service structures [45]. For example, factors such as community support and leadership consistency were identified as important for sustaining delivery of an early years maternal and child health intervention in South Africa [19]. We will examine from interviews and organizational records whether organizations are continuing to deliver PLH after the initial work with program purveyors is completed. We will also explore, using interviews and focus groups, the stakeholder perceptions of barriers and facilitators to continued delivery.

#### (5) What are the costs and resources needed for PLH delivery? 

An essential first step to financial and resource sustainability is understanding the costs in practice and at scale. While it is possible to extrapolate from implementation with smaller numbers of families, costs at scale may differ [49]. There may be economies of scale, for instance, when facilitators deliver multiple rounds of the program after a single training. Therefore, we will explore the costs and resources required for PLH delivery, as well as how the PLH program delivery is currently being funded.

## Study design

This study will use a mixed-methods approach. The research questions, and corresponding implementation phases and data collection methods are outlined in Table 1. The research team will collect primary data in a sub-sample of implementation settings. Data will also be collected by implementing agencies and intervention purveyors as part of the program delivery and may be shared with the research team in a de-identified or anonymized form for secondary data analysis. Although the study timeline is 2020–2024, some of the secondary data has already been collected by implementing agencies and research partners. There can be a tension between pre-specifying research plans and being responsive to dynamic implementation realities. Therefore, further methods and questions may be added, as the study evolves in collaboration with stakeholders.

**Table 1:** Overview of the study data sources

Type of data	Data collectors	Data collection method	Study participants	EPIS phase	Research questions
Primary data	SUPER research team	Interviews and focus groups	Purveyor trainers and program specialists	Preparation Implementation	1,2,4,5
			Donor agency staff	Exploration Preparation Implementation Sustainment	1,2,4
			Local, regional and national policymakers	Exploration Preparation Implementation Sustainment	1,2,4
			Other local stakeholders	Exploration Preparation Implementation Sustainment	1,2,3,4
		Interviews and focus groups	Program coordinators, directors	Exploration Preparation Implementation Sustainment	1,2,3,4,5
			Monitoring and evaluation team of the implementing organization	Implementation Sustainment	2,3,4,5
			PLH facilitators, coaches, trainers	Preparation Implementation Sustainment	2,3,4
		Surveys on organizational characteristics	Program coordinators, directors	Implementation Sustainment	1,2,4
		Budgets, surveys, interviews on costs/resources for delivery	Program coordinators, directors	Implementation Sustainment	5
Primary and secondary data	SUPER research team, implementers, purveyors	Document review of other background materials	Implementing organization, local area	Exploration Preparation Implementation Sustainment	1,2,3,4,5
Secondary data	Implementers (usually collected by program facilitators and other research teams)	Family reports of parenting practices, child behavior, child and caregiver mental health	Families participating in the PLH programs	Implementation Sustainment	3
	Implementers (usually collected by program facilitators)	Family enrolment, attendance, engagement, and dropout		Implementation Sustainment	2,3
	Purveyors	Surveys on the sociodemographic and professional background of staff delivering the program	Facilitators, coaches	Implementation Sustainment	2
		Assessments of competent adherence to the program		Implementation Sustainment	2
		Program documentation, reports	All	Preparation Implementation	1,2,3,4,5

### Study sample

All organizations implementing PLH for Young Children and PLH for Parents and Teens, as well as researchers of formal studies, will be invited to contribute to the study. To allow a more in-depth understanding of our research questions, we will examine three case studies in depth, selected based on variation along the following dimensions: (1) geographical region, (2) level of government involvement in delivery, and (3) number of families reached [18, 50]. Given that the program development and most dissemination have been in the African continent, the case studies will be in Africa.

### Study recruitment and ethical procedures

The study has received ethical approval from the Universities of Oxford (SPICUREC1a__20_015) and Cape Town (PSY2017-040). We have obtained country-level ethics clearance from 15 participating countries, and further, country-level ethics will be collected as the study recruits additional projects. We will seek agreement from each of the organizations involved in the project before approaching individuals within the organization. For primary data collection (Table 1), informed consent will be obtained by the research team. In respect to data shared for secondary analysis, implementation organizations and research teams contributing data to the project are responsible for ensuring that families and staff can consent or opt-out from being a part of the standard monitoring and evaluation data collection, as appropriate. Before any existing data are shared by organizations or investigators, data sharing agreements will be signed; this includes the requirement that partners will anonymize datasets before sharing them.

### Data collection tools and methods

#### Implementation and outcome measures

##### Implementation staff demographic data

Partner organizations and intervention purveyors collect demographic data about PLH facilitators and coaches. Demographic data will include questions on age, gender, marital status, parental status, number and age of children, employment status, previous experience, and educational level. The questionnaire will also assess facilitator/coach self-efficacy, and attitude to corporal punishment.

##### *Facilitator competent adherence*

Facilitator competent adherence to the program can be defined as following the activities outlined in the program manual while also exercising clinical and teaching skills and judgment [51]. Competent adherence to the program will be evaluated using fidelity checklists of session activities and the PLH-Facilitator Assessment Tool (PLH-FAT) [52]. The PLH-FAT is an observation assessment tool used either live during group sessions or with video recordings. The PLH-FAT was developed by the study investigators and program developers to assess the proficiency of program delivery by facilitators as a prerequisite to certification. Seven standard behavior categories outlined in the program manuals are grouped into two scales based on (1) core activities and (2) process skills outlined in the program manuals.

##### Coach competent adherence

Coach competent adherence to the program will be collected using a similar observational assessment tool, the PLH-Coach Assessment Tool (PLH-CAT). This tool assesses the quality of coaching provided to facilitators based on either live observations or video recordings of coaching sessions, including an activity subscale on the discussion of highlights and challenges and similar items for process skills as in the PLH-FAT.

##### Family enrollment and attendance

Enrollment refers to the share of recruited caregivers (and adolescents in PLH for Parents and Teens) who attend at least one session. Attendance refers to the number of sessions, out of the total possible number, attended by an enrolled participant. For the PLH programs, an overall attendance rate is calculated by using both group sessions and home visits, as well as an additional group session rate. Enrollment and attendance data are collected using organizational recruitment lists and attendance registers.

##### Family outcomes

Program purveyors offer organizations implementing PLH a set of family outcome measurement tools. Organizations are encouraged to add these questionnaires to their monitoring and evaluation data collection if they already have a system in place for family data collection, for instance, on HIV risk factors in the context of sub-Saharan Africa. The measures recommended for PLH implementers focus on caregiver and child behaviors and wellbeing (Table 2). The tools were chosen because they assess the core outcomes the PLH programs target and are freely available. PLH for Young Children surveys are parent-reported, while PLH for Parents and Teens has both parent- and adolescent-report versions. Versions of different lengths (short, medium, and long) allow implementers to select the most appropriate version for their needs. Family questionnaires are usually collected by intervention facilitators at the first and last program sessions, with follow-up sessions at home, where possible, in case of missed sessions.

**Table 2:** Family questionnaire measures recommended to implementing partners

Domain	Source	Respondents		
		Parents of young children	Parents of adolescents	Adolescents
Demographics	Selected details (e.g., age, gender, education)	✓	✓	✓
Involved parenting	Alabama Parenting Questionnaire involved parenting subscale [53]	✓	✓	✓
Parental monitoring	Alabama Parenting Questionnaire parental monitoring subscale [53]	✓	✓	✓
Child behavior	Strengths and Difficulties Questionnaire [54]	✓	✓	✓
Child mental health	Strengths and Difficulties Questionnaire [54]	N/A	N/A	✓
Harsh discipline	ISPCAN Child Abuse Screening Tool [55, 56]	✓	✓	✓
Parenting stress	Parenting Stress Scale [57]	✓	✓	N/A
Acceptability of corporal punishment	Multiple Indicator Cluster Survey item [58]	✓	✓	N/A
Parental depression	Centre for Epidemiological Studies-Depression [59]	✓	✓	N/A
Risk avoidance	Risk avoidance planning scale (Developed specifically for Sinovuyo Teen intervention, based on [60]	✓	✓	✓
Economic strengthening	Family financial coping scale (Developed specifically for Sinovuyo Teen intervention, based on [61–63]	✓	✓	✓
Parental support of education	Parental Support for Education Scale [64]	✓	✓	✓

Note: The table presents the most comprehensive version of the questionnaires (“long”). The questionnaires are available on the study’s OSF page

#### Organizational data.

##### Organizational surveys

To characterize the organizations delivering PLH programs, and explore any variation in uptake, implementation, and sustainment between them, we will distribute a short survey to these organizations to capture basic organizational characteristics, such as size and focus of activity.

##### Policies, protocols, and progress reports

Researchers will review locally relevant policies, protocols, and progress reports to identify the context that affects PLH implementation. Information on the implementation process will be extracted from progress reports and other routine documents, such as the implementation readiness assessment, where available. Formal requests will be sent to participating organizations to obtain permission to review relevant documents.

##### Costing questionnaires for organizations

We aim to collect the program set-up and implementation costs from at least the case study countries to examine how much the delivery costs in different contexts. We will review program budgets and collect data directly from the organizations directly about resources involved, based on the Global Health Consortium and J-PAL guidance [65, 66], as well as previous analyses of the PLH for Parents and Teens costs [67].

#### Qualitative data

##### Key informant interviews

Semi-structured interviews will be conducted with representatives of governments, funding agencies, implementing partners, program facilitators, coaches, and trainers. Interviews will be held in person or virtually following interview guides, aiming for approximately 4–8 interviews per implementation site or until saturation is reached.

##### Focus group discussions

Focus group discussions of between 6 and 12 participants will be conducted with PLH program facilitators and supervisors. The number of focus groups per site will be determined by the scale of implementation and heterogeneity of the participants. An experienced moderator will lead the discussions following a discussion guide, assisted by notetakers.

The qualitative data collection will focus on the projects selected as case studies, although it may also include additional sites. Interviews and focus groups will be conducted by experienced interviewers who either speak the same language as the participants or are assisted by an interpreter. Interviews and focus groups will be transcribed. The transcripts will be either written in or translated into English, the main language of analysis. Spot-checking will be done during the translation and transcription phases to ensure consistency and accuracy.

##### Fieldnotes

In addition to interviews and focus groups, fieldnotes will be compiled through stakeholder meetings, such as community of practice meetings. In such meetings, participants involved in the implementation of the programs will be solicited to give an overview of PLH programs in their countries, discuss challenges, and deliberate on possible solutions.

## Analysis plans

Since this is an overarching blueprint, we provide an overview of planned analyses approaches. Where possible, data collection tools, protocols, and detailed plans for specific analyses will be published separately and listed on the project’s Open Science Framework page (https://osf.io/v597r/).

### Quantitative analyses

To address Research Questions 1 and 2 (examining the dissemination and implementation of PLH programs), we will examine the attendance rates and trends among participating families, as well as variation in enrollment, attendance, and program completion. We will also summarize and examine variation in facilitator and coach sociodemographic characteristics, key organizational characteristics, and the relationship of these characteristics to other implementation outcomes, such as facilitator and coach competent adherence to the program, using regression and structural equation models. We will conduct analyses to examine family behaviors and wellbeing before and after participation in the program to address Research Question 3 (impact of PLH at scale on family-level outcomes), using regression-based models. We will also examine variation in family outcomes based on baseline family characteristics and program implementation. We also plan to compare implementation and outcomes associated with different combinations of services as PLH is often delivered alongside other programs. For Research Question 5 (costs and resources), we will summarize the average program costs and their variation.

Techniques such as hierarchical linear modeling and generalized estimating equations will be used to account for the nesting of data—for example, families within areas. Missing data will be addressed using methods such as full information maximum likelihood or multiple imputation [68], or other methods. Reporting will follow STROBE standards [69] where appropriate. Since implementing partners and PLH research teams may have used different measures to assess targeted outcomes, where necessary and possible, data harmonization strategies will be used for analyses involving data from multiple projects.

Where possible, the research team will support implementation teams to lead their own analyses and ensure that data collected in a specific country remains stored there, even if it is shared for additional analysis elsewhere. It is also important to acknowledge that any analysis of routinely collected data, even in relatively high-resource settings, poses challenges, such as with data quality, social desirability, and selection biases [70–72]. In some countries, the study team is providing support to agencies to strengthen data collection and management systems, and a monitoring and evaluation manual is in process of development to support best practices in data collection.

### Qualitative and mixed-method analyses

Thematic analysis [73] will be applied to qualitative data from interviews and focus group discussions to identify patterns within and between case study sites on the dissemination, implementation, and sustainment of PLH programs. Document analysis will be two-phased, combining content and thematic analyses [74]. Relevant information on will be extracted and organized through content analysis guided by the study research questions. The information will then be analyzed thematically. Across our analyses, we will examine how findings vary along key dimensions such as scale of delivery and characteristics of implementing organizations [27, 45, 75].

Reporting will follow the COREQ standards [76]. Summaries of findings will be shared with the research team, participants, and implementing partners for member checking [77]. To support collaboration between researchers, implementers, and other stakeholders [78], we are setting up systems of advisory committees.

Where possible, we will combine multiple data sources to answer research questions. For instance, to help answer Research Question 2 (implementation and adaptation of PLH programs), analysis will draw on the qualitative interviews with facilitators and coaches, and quantitative data on facilitator adherence. We will use the FRAME framework to examine program adaptations [79]. Analyses will include (1) timing and method of adaptation; (2) whether the adaptation was planned or ad hoc; the decision making process for making adaptations; (3) the level and extent of adaptation; (4) whether the adaptation targeted program content, delivery, structure or context of implementation; and (5) how adaptation may have impacted fidelity to core components and program effectiveness.

## Discussion

This project will examine nested information about the community, implementing organizations, types of programs implemented, implementation staff, and the caregivers and children who participate in the PLH programs. The use of multiple data sources and methods is designed to support learning as violence prevention programs are tested, disseminated, and scaled-up. No previous study that we are aware of has collected and examined dissemination and scale-up of a parenting program across multiple LMIC contexts. While this study addresses two specific parenting programs, we anticipate that our findings will be of relevance across the field of parenting, as well as many types of social programming, including those focused on education and violence prevention.

## Supplementary information

**Additional file 1:** TIDieR checklist.

## Data Availability

Key study materials are available on the project Open Science Framework page: https://osf.io/v597r/

## Abbreviations

PLH: Parenting for Lifelong Health
LMIC: Low- and middle-income countries
EPIS: Exploration, Preparation, Implementation and Sustainment (framework)
WHO: World Health Organization
